# A *Caenorhabditis elegans* Locomotion Phenotype Caused by Transgenic Repeats of the *hlh-17* Promoter Sequence

**DOI:** 10.1371/journal.pone.0081771

**Published:** 2013-11-28

**Authors:** Randy F. Stout Jr, Vladimir Grubišić, Vladimir Parpura

**Affiliations:** 1 Department of Neurobiology, Center for Glial Biology in Medicine, Atomic Force Microscopy & Nanotechnology Laboratories, Civitan International Research Center, Evelyn F. McKnight Brain Institute, University of Alabama, Birmingham, Alabama, United States of America; 2 Department of Biotechnology, University of Rijeka, Rijeka, Croatia; Inserm U869, France

## Abstract

Transgene technology is one of the most heavily relied upon tools in modern biological research. Expression of an exogenous gene within cells, for research and therapeutic applications, nearly always includes promoters and other regulatory sequences. We found that repeats of a non-protein coding transgenic sequence produced profound changes to the behavior of the nematode *Caenorhabditis elegans*. These changes were produced by a glial promoter sequence but, unexpectedly, major deficits were observed specifically in backward locomotion, a neuron-driven behavior. We also present evidence that this behavioral phenotype is transpromoter copy number-dependent and manifests early in development and is maintained into adulthood of the worm.

## Introduction

Phenotypic effects of transgenes driving expression of various fluorescent reporter proteins are common and have been attributed to several factors, such as over expression of a foreign protein within cells, the site of transgene integration, or transfection vector [1-3]. However, possible effects of the trans-promoter sequence used to direct expression of such transgenes have received little attention although there are indications that it may influence cellular processes in unexpected ways [4]. 

The most widely used method to introduce transgenes into the popular model organism *C. elegans* involves injecting plasmid DNA directly into the gonad syncytium of adult worms [5,6]. This procedure produces an array of repeats of the injected DNA. The repeat number within an array varies between individual strains and depends on the concentration of DNA injected along with other factors. These repetitive sequences are transmitted as extrachromosomal arrays to offspring with mosaicism across the cells within individual worms and variable transmission to progeny [6]. To overcome these issues, arrays can be integrated randomly into the endogenous genomic DNA through several methods, including gamma irradiation [6]. Once transgenic arrays are integrated they are generally transmitted in a similar way as endogenous genes [6].

Highly repetitive transgene sequences in extrachromosomal or integrated form are epigenetically marked and localized to the periphery of the nucleus and incompletely silenced [7,8]. Transgenic arrays containing cell-specific promoters are in widespread use as research tools, although cellular responses to foreign repetitive elements are not fully understood. 

The *hlh-17* promoter (*Phlh-17*) has been used as a marker for the cephalic sensilla sheath (CEPsh) glial cells [9-14]. It drives strong expression of fluorescent reporter proteins in the four CEPsh glial cells in the head region of *C. elegans* from the embryo to the adult stage, and in some transgenic strains, weak transient expression in other cells [10,11]. We initially identified a variably penetrant, but stereotypic behavioral phenotype in some worm lines carrying transgenic constructs consisting of the transgenic *hlh17* promoter (t-*Phlh-17*) sequence driving glial cell specific expression of a fluorescent protein. We conducted tests to characterize this phenotype and to determine the cellular mechanistic basis. We found that the phenotype was caused by sequences in the *hlh-17* promoter instead of those coding for protein. We also present results strongly indicating that this transgene-induced behavior is transpromoter copy number-dependent and is produced early during development of the nervous system and is maintained into adulthood of the worm.

## Materials and Methods

### Transgenic construct production

All plasmids made in the laboratory for transgenic VPR worm line production in this study and their sequences are available upon request. These plasmids are summarized in Table 1, along with additional, previously published plasmids available from original sources. All plasmids contain the unc-54 3’untranslated region sequence (UTR) following the promoter or the gene, with the exception of pDP#MM016B which contains the *unc-119* 3’ UTR. We list the plasmids below with their transpromoter and transgene disclosed in brackets associated with the plasmid name.

**Table 1:** Summary of plasmids by name, background vector, their promoter and gene content, and location of gene expression in transgenic *C. elegans* cells.

**Plasmid**	**Background Vector**	**Promoter**	**Gene**	**Expression**
pRSG	pPD95.69	*Phlh-17*	*GFP*	CEPsh glia
pRSRXP2	pPD95.69	*Phlh-17*	*DsRedExpress2*	CEPsh glia
pRSR2*	pPD95.69	*Phlh-17*	*monomericDsRed*	CEPsh glia
pRSV2	pPD95.69	*Phlh-17*	*none*	NA
pDP#MM016B **	pBluescript II KS(-)	*Punc-119*	*unc-119*	most neurons
pU54mCh **	pPD30.38	*Punc-54*	*mCherry*	muscle cells

Abbreviations: CEP, cephalic sensilla; CEPsh, CEP sheath.

* used pRSDAR [*Pdat-1::monomericDsRed*] as template (see materials and methods)

** previously published: pDP#MM016B [17] and pU54mCh [24].

#### pRSG [*Phlh-17::GFP*]

We copied the *hlh-17* promoter (as originally described in [10]), from genomic DNA with a pair of primers (forward, *hlh-17* BamHI: ggccaggatccgaacagcttagctatttcgt; and reverse, *hlh-17 Xmal*: ctttggccaatcccccgggtccatgactgg) which creates BamHI and XmaI restriction enzyme recognition sites on the 5’ and 3’ ends of a 2.5 kbp fragment 118 bp before the translation start site of the *hlh-17* gene [10,11]. The promoter was inserted into *C. elegans* vector pPD95.69 (courtesy of A. Fire, Stanford University, Palo Alto, CA). Since pPD95.69 contains *GFP* followed by the *unc-54 3’UTR*, by cutting pPD95.69 and the PCR product with BamHI and XmaI, followed by ligation, the resulting pRSG contains *Phlh-17::GFP*::*unc-54 3’UTR*. For simplicity, in subsequent construct descriptions we omit referral to *unc-54 3’UTR*, unless necessary. The following plasmids containing the *Phlh-17* used in reported work are modifications of this plasmid. 

#### pRSRXP2 [*Phlh-17::DsRedExpress2*]

PCR was performed with a pair of primers (forward: aacacgatgataacccgggtatggccacaacc; and reverse: tagagtcgcggccgctacaggaacag) and pIRES2-DsRedExpress2 (Clontech, Mountain View, CA) as template. The PCR product containing *DsRedExpress2* was inserted into a modified and previously published version of pRSG, pRSFX4 [15], using digestion of product and vector with XmaI and NotI, followed by ligation. Thus, the resulting pRSRXP2 contains *Phlh-17* which drives expression of the *DsRedExpress2* coding sequence.

#### pRSDAR [*Pdat-1::monomericDsRed*]

The *GFP* in pRB490 (pPD95.73 backbone vector containing *Pdat-1::GFP*; kindly provided by R. Blakely, Vanderbilt University, Nashville, TN) [16] was replaced by *monomericDsRed* from pDsRed-Monomer-C1 (Clontech, Mountain View, CA) by cutting both plasmids with the enzymes AgeI and EcoRI, followed by ligation. The resulting pRSDAR was used as a template for production of pRSR2 below. 

#### pRSR2 [*Phlh-17::monomericDsRed*]

PCR was performed with a pair of primers (forward: gccggatccccgggattggccaaggacc; and reverse: cgtacggccgactagtaggaaacag) and pRSDAR as template to amplify a fragment containing monomericDsRed with the *unc-54 3’UTR*. The resulting PCR product and pRSG were trimmed/cut with XmaI and SpeI restriction enzymes (removing the *GFP* and *unc-54 3’UTR* from pRSG) and the trimmed *monomericDsRed::unc-54 3’UTR* fragment was inserted by ligation. The resulting pRSR2 contains the *Phlh-17* which drives expression of *the monomericDsRed* coding sequence.

#### pRSV2 [*Phlh-17p::none*]

We made this plasmid as pRSG, but with pPD95.69 with its *GFP* coding sequence removed. This removal was done by first, treating pPD95.69 with enzymes BamHI and SpeI which removed the *GFP* with *unc-54 3' UTR* , and then, the *unc-54 3' UTR* was re-inserted after obtaining it by BamHI and SpeI restriction of pBY103 [kindly provided by M. Maduro, University of California, Riverside, CA [17]]. The resulting pRSV2 contains only *Phlh-17* with the downstream *unc-54 3' UTR* sequence, but lacking a transgene.

### Transgenic line production and selection

Table 2 provides a summary of worm lines used in this work. All transgenic VPR lines made in the laboratory for this study are available upon request; new lines have been deposited in the Caenorhabditis Genetics Center (CGC; University of Minnesota, Minneapolis, MN; http://www.cbs.umn.edu/CGC; funded by the NIH National Center for Research Resources). Transgenic lines were constructed by germline transformation after microinjection [5] of plasmids (75 ng/µL with the exception of the pRSV2 at 100 ng/µL, pU54mCh at 50 ng/µL or 150 ng/µL) into N2 worms. Transgenic arrays were integrated by gamma irradiation. These integrated lines were backcrossed 2 times to N2 unless otherwise stated. Worms were maintained at 20°C on standard nematode growth medium/agar (NGM) seeded with OP 50 strain *E. coli* [18]. We list transgenic worm lines with their transgenes carried as extrachromosomal (*Ex*) or integrated (*Is*) arrays reported in parentheses within which trans-promoter/gene are in brackets. MS839 and UL1713 lines were made from worm strains rooted in the N2 (non-transgenic, Bristol strain) background, while all other lines were made directly into the N2 strain.

**Table 2:** Description of utilized *C. elegans* strains listing the plasmid(s) used in the production of each line, if the transgene is carried as an extrachromosomal (*Ex*) or integrated (*Is*) array, and the presence of the ventral coiler phenotype (VCP).

**Strain**	**Plasmid(s)**	**Integrated**	**Genotype**	**VCP**
N2	None	NA	non-transgenic Bristol N2	N
VPR839	pRSG + pDP#MM016B	Y	*N2*, *irIs67*[*Phlh-17::GFP + unc-119(+*)]	N
VPR127	pRSG	Y	N2, *vprIs127* [*Phlh-17::GFP*]	Y
VPR157	pRSG	Y	N2, *vprIs157* [*Phlh-17::GFP*]	Y
VPR128	pRSXP2	Y	N2, *vprIs128* [*Phlh-17::DsRedExpress2*]	Y
VPR156	pRSR2	Y	N2, *vprIs156* [*Phlh-17::monomericDsRed*]	Y
VPR160	pRSV2 + pU54mCh	N	N2, *vprEx160* [*Phlh-17::none + Punc-54::mCherry*]	Y
VPR163	pU54mCh	N	N2, *vprEx163* [*Punc-54::mCherry*]	N
*VPR168	NA	N	N2, *wyEx915* [*Phlh-17::mCherry + Punc-122::GFP*]	N
**VPR108	NA	Y	N2, *vprIs108* [*Phlh-17::GCaMP2.0 + Phlh-17::mCherry*]	Y
**U1713	NA	N	*unc-119(ed3) III, leEx1713 [Phlh-17::GFP + unc-119(+)]*	N

Note: Plasmid descriptions are available in Table 1; NA, not applicable. Locomotion phenotype is presented in the binary form (N, Normal vs. Y, VCP), coded based on the average VCP proportion cut-off set at 0.1. * VPR168 was obtained by crossing previously published TV2394 [9] with N2, followed by progeny selection. ** previously published: UL1713 [12] and VPR108 [13,15].

#### VPR839 (*irIs67[Phlh-17::GFP + unc-119(+*)])

This line was produced by backcrossing MS839 line 4 times to N2 strain to eliminate *unc-119* (*ed4*) *III* mutation. MS839 (*unc-119* (*ed4*) *III, irIs67*[*Phlh-17::GFP + unc-119(+*)]) line was produced by co-microinjection of plasmids pRSG and pDP#MM016B into *unc-119* (*ed4*)*III* worms (kindly provided by M. Maduro) [17] and then screened for rescue of the *unc*-119(+) phenotype, followed by transgene array integration. 

#### VPR127 (*vprIs127 [Phlh-17::GFP*]) and VPR157 (*vprIs157 [Phlh-17::GFP*])

These lines were produced by microinjecting N2 worms with the same plasmid (pRSG) as that of MS839. They carry unique arrays as they were sourced from different injected N2 parent worms. 

#### VPR128 (*vprIs128 [Phlh-17::DsRedExpress2*]) and VPR156 (*vprIs156 [Phlh-17::monomericDsRed*])

N2 worms were microinjected with either pRSXP2 or pRSR2 plasmid, respectively, and selected by expression of red fluorescent protein in the CEPsh cells.

#### VPR160 (*vprEx160 [Phlh-17::none + Punc-54::mCherry*]) and VPR163 (*vprEx163 [Punc-54::mCherry*])

N2 worms were microinjected with pRSV2 and/or pU54mCh (an injection marker). Lines were individually selected by expression of red fluorescent protein in muscle cells.

VPR168 (*wyEx915 [Phlh-17::mCherry + Punc-122::GFP*]) was generated by crossing TV2394 with N2, which was followed by progeny selection. Worms containing *wyEx915* [*Phlh-17::mCherry + Punc-122::GFP*] but lacking *Punc-122::RFP* array were selected. 

#### TV2394 (*wyEx915 [Phlh-17::mCherry + Punc-122::GFP*], *[Punc-122::RFP]); asterisks indicates unnamed array

This line was generously provided by D.A. Colόn-Ramos (Yale University, New Haven, CT) which was produced as previously described [9]. Due to injection of pDACR (backbone vector pPD49.26) encoding *Phlh-17::mCherry*, CEPsh glial cells of this line express mCherry. Additionally, *Punc-122::GFP and Punc-122::RFP* drive expressing of these fluorescent proteins in coelomocytes [for reporter expression driven by *Punc-122* see 19; information of coelomocytes available at http://www.wormatlas.org/hermaphrodite/coelomocyte/Coelomoframeset.html]. 

#### VPR108 (*vprIs108 [Phlh-17::GCaMP2.0 + Phlh-17::mCherry*])

We previously produced this line [13,15] by co-microinjection of plasmids encoding *Phlh-17::GCaMP2.0* and *Phlh-17::mCherry*, with array integration, followed by backcrossing to N2 four times. 

UL1713 (*unc-119*(*ed3*) *III, leEx1713*[*Phlh-17::GFP + unc-119*(+)] was generously provided by the CGC on behalf of A. J. Walhout’s laboratory at the University of Massachusetts Medical School, Boston, MA (line produced by I. A. Hope’s group at the University of Leeds, UK). This line was produced by particle bombardment as previously described in the supplementary data of [12]. It should be noted that the *Phlh-17* used in this line, due to its 5’ end truncation, is ~2 kbp in length, as opposed to the 2.5 kbp length used in other plasmids/transgenics for this work.

Transgenic lines were selected with a macro zoom microscope (MVX10, Olympus) equipped with transmitted-light (30 W Halogen) and wide-field fluorescence (100 W Xenon arc) illumination along with standard GFP, DsRed, and dual GFP-DsRed filter sets (Chroma Technology). Selecting for Ventral Coiler Phenotype (VCP) severity testing and assigning a blind key (with number designation from a list of random four digit numbers), was a two-step procedure: i) prior to testing we picked individual hermaphrodite young adult worms, which were the F1 generation of a single hermaphrodite, heterozygous for the *vpr156* array, and then ii) post testing, individual worms were allowed to have self-fertilized offspring with the genotype of the F1 generation assessed based on F2 assortment and then matched back to the pretesting selection key.

### Behavior testing

Behavior testing was carried out at 20°C on growth plates using transmitted light images from a macro zoom microscope equipped with: i) a Gigaware® 2.0MP webcam (Ignition L.P.) mounted to an ocular [20] and driven by Webcam Companion 3 software; and ii) a top port mounted CoolSNAP *HQ* ^2^ camera (Photometrics) driven by Metamorph™ imaging software ver. 7.0 (Molecular Devices Inc.). The invertebrate *Caenorhabditis elegans* is not covered by the Guide for the Care and Use of Laboratory Animals of the National Institutes of Health and an animal use protocol and Institutional Animal Care and Use Committee approval are not required for work involving this invertebrate species.

#### A): VCP Proportion

Each worm was tested in 6 trials, each trial containing sequential testing for forward and backward crawling. To assure viability of each worm, forward crawling was induced by touching the posterior quarter of the worm’s body once with an eyebrow hair attached to a toothpick [21]. Worms that were able to crawl forward were then tested on backwards crawling by touching the anterior quarter of the body repeatedly until the worm crawled backward for one body length (Movie S1) or one of the following criteria were met which constituted exhibition of the VCP for that trial (Movie S2): i) the tail touched any part of the worms body, or ii) the worm initiated backward movement and the anterior section of the body continued moving while the tail remained frozen in an abnormal curved position (tail paralysis). The VCP proportion for each worm was calculated as the number of failures to crawl backward in each set of six trials. Each line was tested from at least 3 separate plates with at least 6 worms per plate. In Table 2 we present the locomotion phenotype in binary form (Normal vs. VCP). This binary categorization was obtained by setting the cut-off value of the average VCP proportion at 0.1. If the average VCP proportion for a particular strain was < 0.1, the binary code was set to “Normal”, otherwise it was set to “VCP.”

#### B): VCP severity

Each selected worm was filmed and resulting video clips were scored blind to genotype on a counting number scale ranging from 1 to 10 (1 being completely normal backwards crawling, while 10 being severe VCP: a tight curl of the tail immediately or “freezing” upon initiation of backward movement) for severity of the VCP (Movies S3 and S4). After scores were assigned to each worm they were matched back to a key containing the genotype, determined as described above. 

### Morphological assessment of transgenics

For morphological assessment of transgenic worms, individual worms were immobilized by sodium azide solution (20 mM), deposited onto a glass coverslip and imaged with a 20 x oil immersion objective (0.80 NA) of a FluoView FV 300 (Olympus) confocal laser scanning microscope controlled by FluoView 5.0 software. We used an Argon ion laser (10 mW at 488 nm) for excitation of GFP, and a HeNe ion laser (1 mW at 544 nm) for monomericDsRed excitation, while respective emission was collected at FITC (green channel) and TRITC (red channel) settings. The entire width of each worm was imaged by serial sagittal z-plane section image scans and the resultant image stacks were processed as maximum projection reconstructions using Metamorph™. 

### Single worm genomic DNA extraction and DNA copy number (CN) assessment

A single adult worm was picked from a standard NGM plate, isolated on a 1.5 % agarose plate for 5-10 min and then placed into 10 µL of lysis buffer (50 mM KCl, 10 mM Tris pH 8.2, 2.5 mM MgCl_2_ and 0.45% Tween-20) containing 1 mg/mL of protease K. The lysate was frozen and maintained at -80°C for at least two hours and then sequentially incubated for 1.5 h at 60°C and 15 min at 90°C. The lysate was diluted 5-10 times with the DNA grade water and used as template for DNA amplification. The quality of DNA extraction for each run was confirmed by standard PCR prior to submitting DNA to real-time quantitative PCR (RT-qPCR), which was used to obtain the CN. 

All primer pairs were designed to have products of ~250 bp targeting [chromosome (Ch) numeration with the target locus indicated as a base pair range]: (i) a non-coding section of chromosome 1 (Ch1: 14866251-14866278; forward, tggaagatgttgaagtcgataacgaatg, and reverse, tgcacaccgccacgttctcac) which served as the normalizer; (ii) the *hsp-1* gene promoter region (*Phsp-1*; Ch4: 17277751-17277780; forward, gataccgtctagttttgacaggtgttcaac; and reverse, ttctcattcctaatttcccgacctttc), which served as the CN control; and (iii) the 3’ region of the *hlh-17* promoter (*Phlh-17*; Ch4: 16254104-16254127; forward, acccactcgccaccactcattatc; and reverse, cgctgactcgtctggagaaagtagaac). Targeting was aided by the Basic Local Alignment Search Tool (BLAST) database WS229 and BLASTIN 2.2.17 [22]. 

The validity of the primers was confirmed by regular PCR and the Roche FastStart PCR Master mix (Cat. No. 04710436001) on genomic DNA extracts. Each and all primer pairs showed single band products ~ 250 bp with excellent PCR efficiency (Ch 1, 2.01±0.01; *Phlh17*, 2.01±0.03; and *Phsp-1*, 2.04±0.09; mean+sem). A Roche LightCycler® 480 Real-Time PCR System with 384-well plates and Roche LightCycler® 480 SYBR Green I Master mix (Cat. No. 04707516001; which conveniently contains the same DNA polymerase as the PCR master mix utilized in regular PCR) were used for RT-qPCR. Each sample was run in triplicates. Two types of negative controls (water and lysis buffer as “templates”) were run in duplicates for every primer pair. RT-qPCR results were subjected to Crossing point (Cp) analysis; the PCR cycle at which the fluorescence of a sample rises above the background fluorescence was obtained by the 2^nd^ derivate Max method (LightCycler® 480 Instrument Operator’s Manual Software Version 1.5). Sample Cp values were not included in analysis if: (i) they had more than one melting temperature indicating primer dimers; (ii) the Cp value detected was >40; or (iii) negative controls were positive and the difference in Cp values between the sample and control was < 7. CN values were calculated for each primer pair and each worm genomic DNA sample using the formula:

$Target\;\;CN\;=\;PCR\;\;efficiency{\;}^{\left(Normalizer\;Cp\;-\;Target\;Cp\right)}$ (Eq. 1).

The *Phsp-1* primer pair, targeting the single locus on the Ch4, served as a control, showing accordingly the *Phsp-1* CN of 1.1±0.1 in N2, which was not statistically different between various strains (VPR839, UL1713, VPR127, VPR160, VPR156 and VPR108; p = 0.5238, Kruskal-Wallis one way ANOVA). Note that the *Phlh-17* primer pair had targets at two additional endogenous locations in the worm genome (Ch^3^: 5360761-5360738 and ChX: 17697513-17697490); due to this additional endogenous targeting, the raw *Phlh-17* CN of 2.9±0.3, as expected, was obtained using the N2 worms. The CN values we report using this primer set were in good agreement with those obtained using an additional primer set targeting the 5’ region of the *Phlh-17* (Ch4: 16252922-16252945; forward, aatgcttcctgctccatctgttgc; and reverse, agcaagcaagggttgctattccag). Thus, this dual survey of the 5’ and 3’ regions of the *Phlh-17* promoter increases the likelihood that CN values represent a reflection of the entire promoter, rather than that of incomplete fragments of injected plasmids.

### *In silico* motif identification

Sequences were examined and visualized by the software Geneious v6.1.5, Biomatters Ltd. Motifs were identified using a version of the fuzznuc tool which is part of the EMBOSS 6.5.7 software suite [23].

### Statistics

We used the GB-Stat 6.5 software (Dynamic Microsystems Inc., Silver Spring, MD). In order to use tests with the highest statistical power, we performed data transformation when needed to achieve normal distributions and homoscedasticity (for multiple comparisons). We used non-parametric tests if these parameters could not be corrected, even for a single variable.

## Results

**Figure 1:**
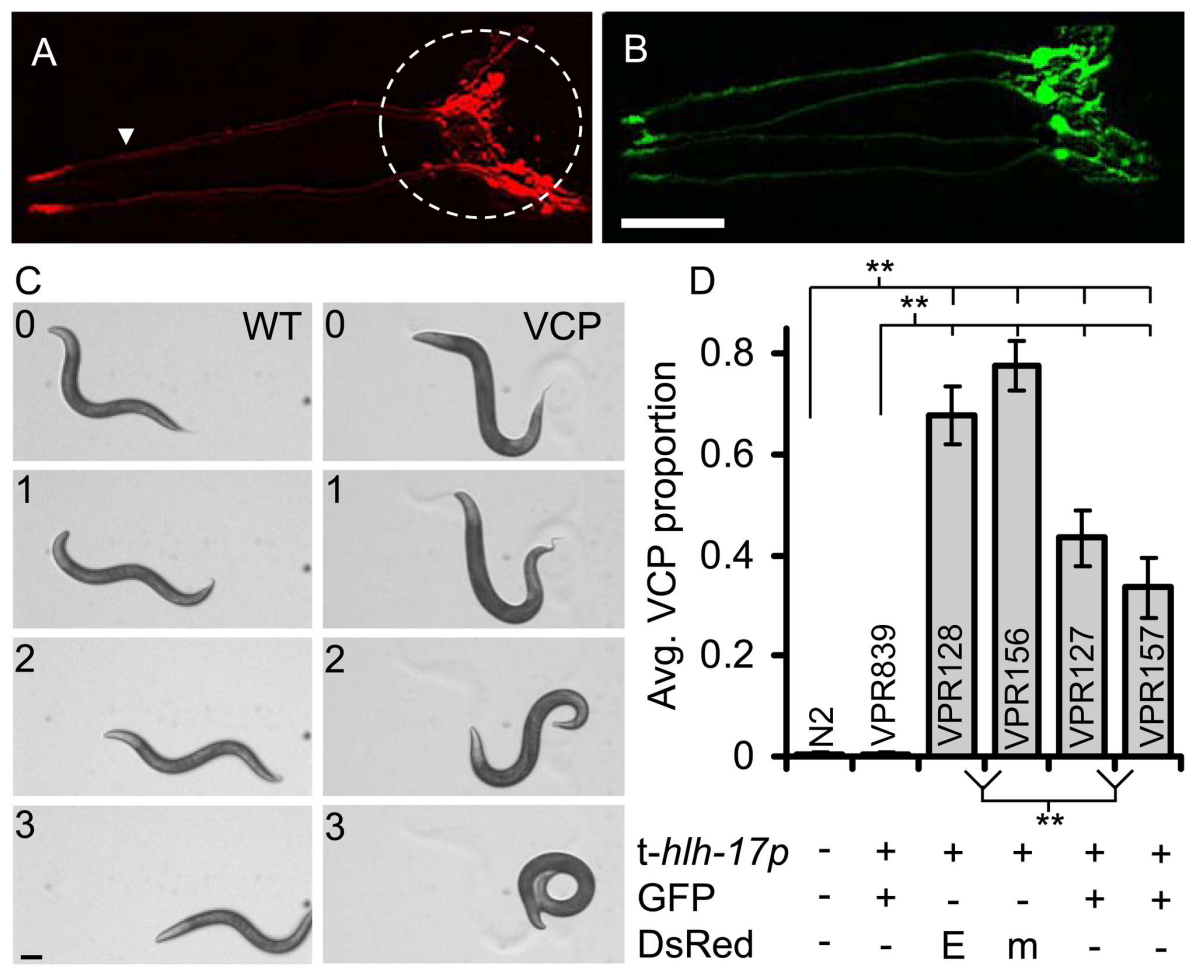
A subset of *C. elegans* lines carrying transgenic arrays containing the trans-*hlh-17* promoter (t-*Phlh-17*) to drive expression of fluorescent proteins in the CEPsh glial cells display a ventral coiler phenotype (VCP) during backward movement. A-B) Transgenic worm lines expressing fluorescent proteins in the CEPsh glial cells driven by the t-*Phlh-17*. Confocal images show anterior, head, portion of worms. (A) VPR156 shows cytosolic monomericDsRed expression. Dashed circle indicates the CEPsh glial cell bodies and membrane extensions. The arrowhead indicates the thin processes emanating to the anterior sensory structures. (B) VPR839 shows similar GFP expression in the CEPsh glial cell. Scale bar, 20 μm. (C) Image series depicting normal spontaneous reverse movement (WT, left column) and spontaneous VCP in line VPR156 (VCP, right column). Worms are shown crawling with right lateral side of the body on the agar surface. Numbers indicate time in seconds. Scale bar, 100 μm. (D) Proportion of trials in which the touch-induced VCP was displayed by N2 (non-transgenic, Bristol strain) and t-*Phlh-17* transgenic (VPR839, VPR128, VPR156, VPR127, and VPR157) strains. The matrix below the graph indicates the composition of transgenes in each line (E, DsRedExpress2; m, monomericDsRed). Bars represent means + sem; n=53 for all groups; ** indicates a significant difference at p<0.01, Kruskal-Wallis one-way ANOVA (KWA) followed by Newman-Keuls multiple comparisons test (MCT).

We identified abnormal locomotion in the form of the ventral coiler phenotype (VCP) in worms of two transgenic *C. elegans* lines. These lines both carry genome integrated arrays, one *vprIs128* (*Phlh-17::DsRedExpress2*) and the other v*prIs156* (*Phlh-17::monomericDsRed*), and thus expressing red fluorescent proteins (DsRedExpress2 or monomericDsRed, respectively) in CEPsh glial cells, driven by the transgenic *hlh-17* promoter (t-*Phlh-17*) (Figure 1A). These lines coil with the ventral side of the body to the interior of the coil only when undergoing backward movement (see Table 2 for strain descriptions). This behavior can occur spontaneously (Figure 1 C, VCP column) or in response to mechanical stimulation to the anterior of the worm (Figure 1D; also see Movies S1 and S2). We quantified the proportion of times (out of 6 trials) that individual worms exhibited the VCP. VPR128 and VPR156 worms failed to crawl backwards with an average VCP proportion of 0.67+0.06 and 0.78+0.05 (mean+sem), respectively. This was significantly higher than the proportion in N2 (Bristol strain) control worms (average VCP proportion= 0.003+0.003) (Figure 1 D) which displayed normal backward crawling (Figure 1 C, WT column). However, we did not observe the VCP in the VPR839 line which carries a transgenic construct containing the t-*Phlh-17* driving the expression of green fluorescent protein (GFP) in CEPsh glial cells (Figure 1 B,D).

Since the gene encoding DsRed is substantially different in sequence from that of GFP, the data available from these lines might be taken as an indication that the VCP could be reporter protein specific. Consequently, we examined the two additional lines, VPR127 and VPR157 that carry integrated copies of the t-*Phlh-17::GFP* array, which exhibited the VCP with proportions significantly higher than those observed in N2 and VPR839 worms, albeit smaller than those of DsRed variant-expressing worms (Figure 1 D). The latter fact should not distract from the major finding: the presence of the VCP in worms expressing either GFP or DsRed variants. In further support of this notion that the VCP is not fluorescent reporter protein specific, when we tested worm lines in which the t-*Phlh-17* drives the expression of GFP (UL1713; n=18; average VCP proportion= 0.08+0.03) [12], mCherry (VPR168; n=53; average VCP proportion= 0.08+0.03) [9] and GCaMP2.0+ mCherry (VPR108; n=18, average VCP proportion= 0.39+0.09) [13,15], we found them displaying the VCP at various proportions; note that when locomotion phenotype is presented in the binary form, based on the arbitrary cut-off (see material and methods), only VPR108 would be classified as VCP (Table 2). Furthermore, the variability in the VCP proportion recorded from different genome integrations of the same transgene (*Phlh-17::GFP*) points to level of protein expression and/or copy number (CN) of the t-*Phlh-17* itself as the cause of the VCP. 

**Figure 2:**
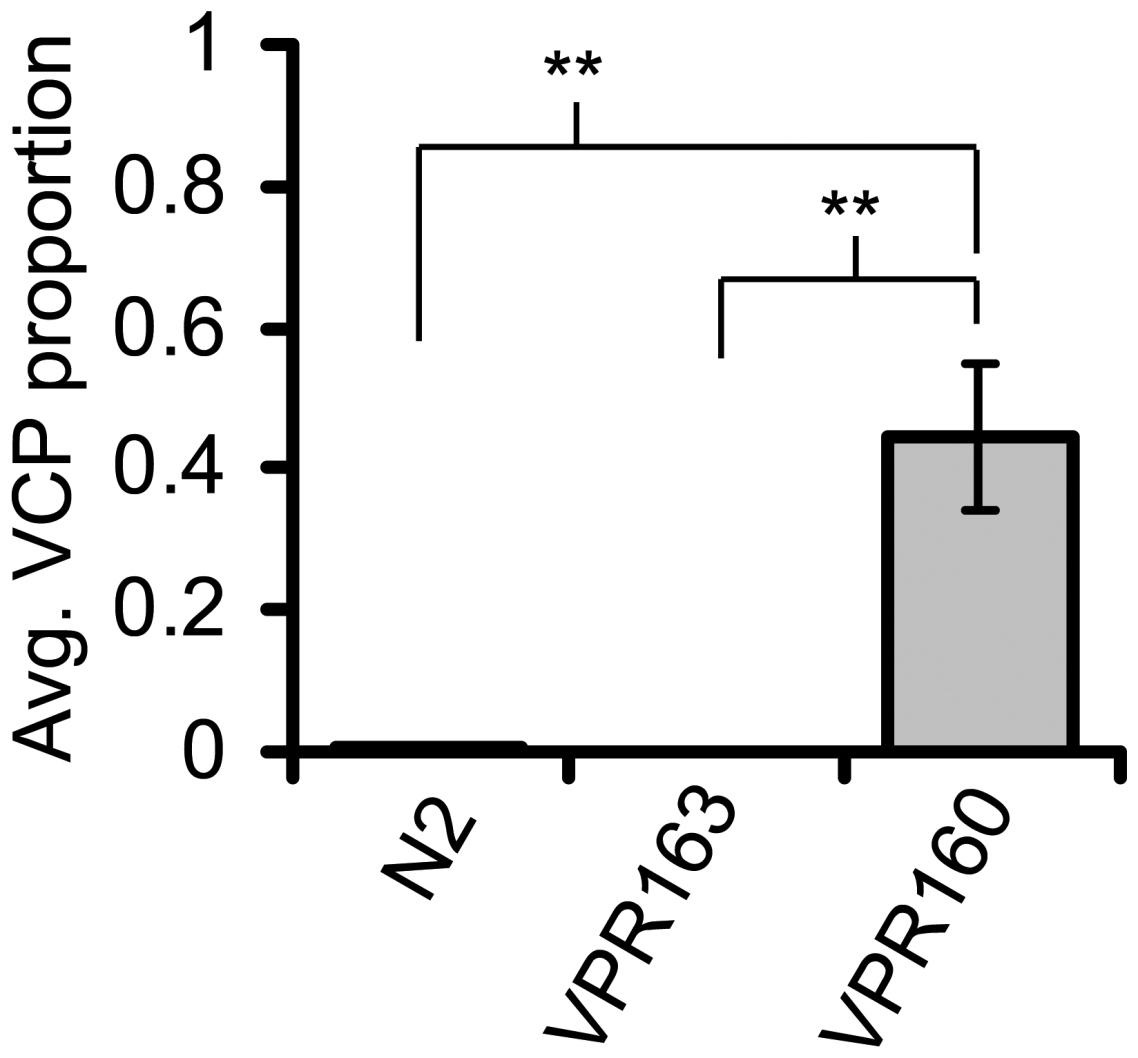
The t-*Phlh-17* produces the VCP without driving fluorescent protein expression. Average proportion of trials in which the VCP was displayed by strains N2, VPR163 (*Punc-54::mCherry* alone; n= 21) and VPR160 (*Phlh-17::none* + *Punc-54::mCherry*; n= 21). Bars represent means + sem. ** p<0.01, KWA followed by Dunn’s MCT.

To test if over-expression of protein by the t-*Phlh-17* caused the VCP, we used a transgenic construct that included the t-*Phlh-17* but lacked downstream protein expression. Here, we made the transgenic line VPR160, with a similar construct as one used to make strain VPR839, but lacking the GFP coding sequence downstream of the t-*Phlh-17*. To report on success of transgenic line production and achieve their selection based on red fluorescence, we co-injected *Punc-54::mCherry* to drive reporter expression in muscle cells, as previously described [24]. As a control we made the VPR163 line expressing only this red muscle marker, which did not cause the VCP (Figure 2). However, there was a robust display of the VCP in the VPR160 strain (Figure 2). Taken together, protein expression driven by the t-*Phlh-17* is not necessary for the VCP. At this juncture, it should be noted that the same downstream sequences (fluorescent protein coding regions and the *unc-54 3’UTR*) are present in strains that show the VCP (e.g.,VPR160) as well as those strains that do not show the VCP (e.g.,VPR163). These results indicate that sequences in the plasmids other than the t-*Phlh-17* are likely not the cause of the VCP. Gene disruption of endogenous genes by the integration of the transgene cannot be the cause of the VCP since VPR160 carries an un-integrated, extrachromosomal array and worms from this line display the VCP.

**Figure 3:**
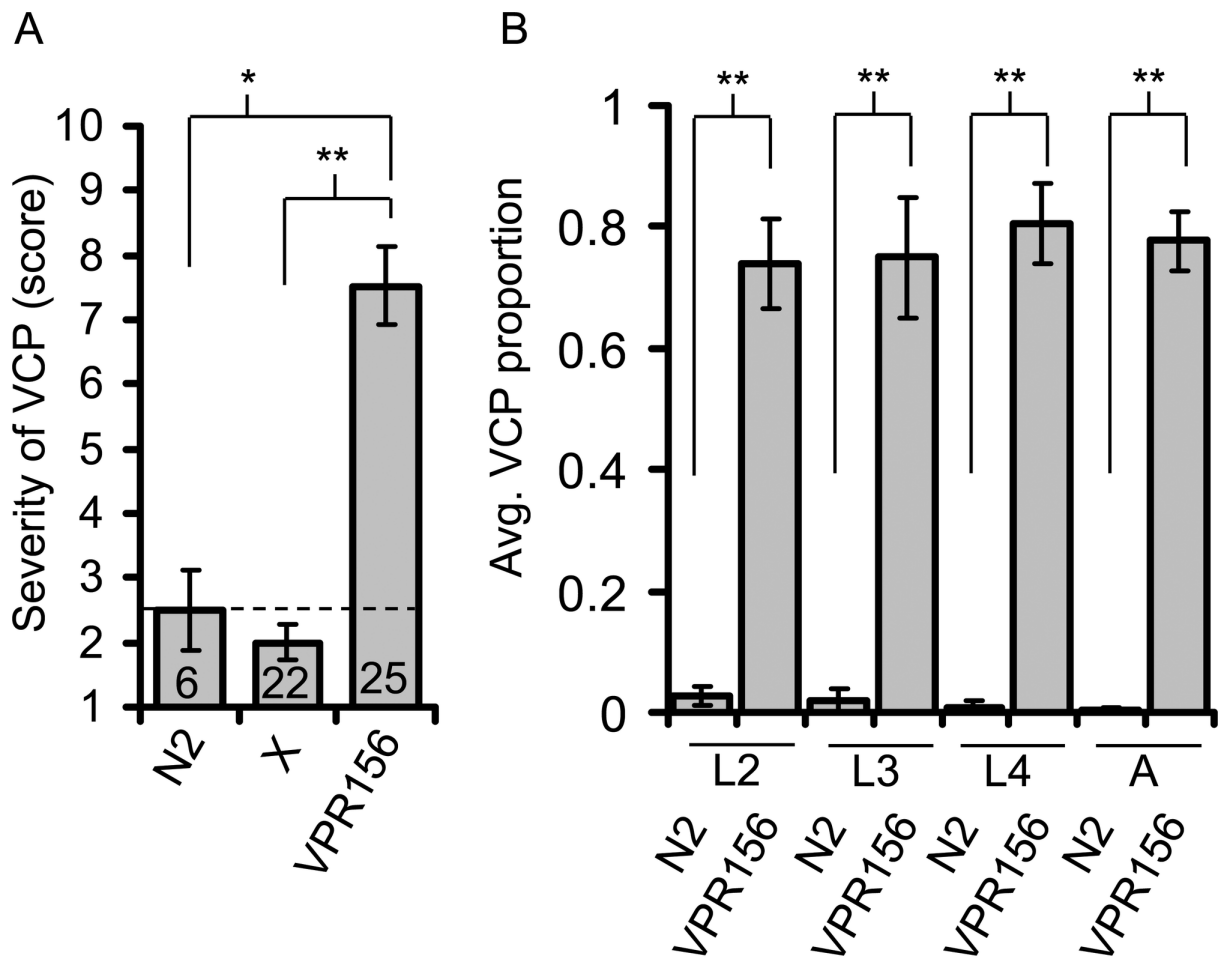
The severity of the VCP depends on copy number of the trans-promoter and is maintained from early larval stages through adulthood. (A) Averaged VCP severity rating assigned to worms (parental N2 and VPR 156 lines, as well as their cross, X, heterozygous for the integrated transgenic array) based on a 1-10 counting number scale (1, normal backward crawling; 10, full VCP). While VPR156 line shows VCP (Movie S2), its cross to N2 does not (Movie S3). Dashed line indicates baseline score for N2. Number of worms tested for each category is shown within each column. Groups were compared by KWA followed by Dunn’s MCT; **p<0.01, * p<0.05. (B) The VCP is maintained from early larval stages through adulthood. Average proportion of trials in which the VCP was displayed by N2 and VPR156 lines at various life stages. L, larval stages 2-4; A, adult. Note that L1 worms were not tested due to their size. Data for adults is sourced from Figure 1D (n=53); n=18 for both genotypes for all other life stages shown. Groups at each life stage were compared by Mann-Whitney U-test; ** p<0.01. Bars in A and B represent means + sem.

Having determined that t-*Phlh-17* itself is the cause of the VCP, we next tested if copy number of the promoter (CN) is relevant for the severity level of the phenotype. Since CN of an integrated transgene is normally stable between generations, we were able to increase and decrease the CN through breeding. We also implemented a more sophisticated analysis of touch-induced back crawling, based on scoring with a counting number scale (1-10; 1, normal, while 10, full VCP), in order to assess the severity of the VCP (Movies S3 and S4). To determine if decreasing CN reduces phenotype severity, the VPR156 line, which has high VCP severity, was crossed to N2. The VCP required a double dose of the *vprIs156* array since, since the cross, which is heterozygous for the array, lacked the VCP and behaviorally was statistically indistinguishable from N2 on average (Figure 3 A). Thus, the CN of t-*Phlh-17* can modulate the extent of VCP display/severity.

In all experiments presented thus far we used adult worms. To assess whether the VCP might be developmentally regulated, we compared the VPR156 line showing severe VCP with non-transgenic N2 worms at various life stages (larval 2-4 and adult). The VCP was seen at all stages in VPR156, while N2 worms showed normal locomotion (Figure 3 B). Thus, the VCP appears to be a developmental defect that occurs early and is maintained into adulthood as opposed to a neurodegenerative effect.

**Figure 4:**
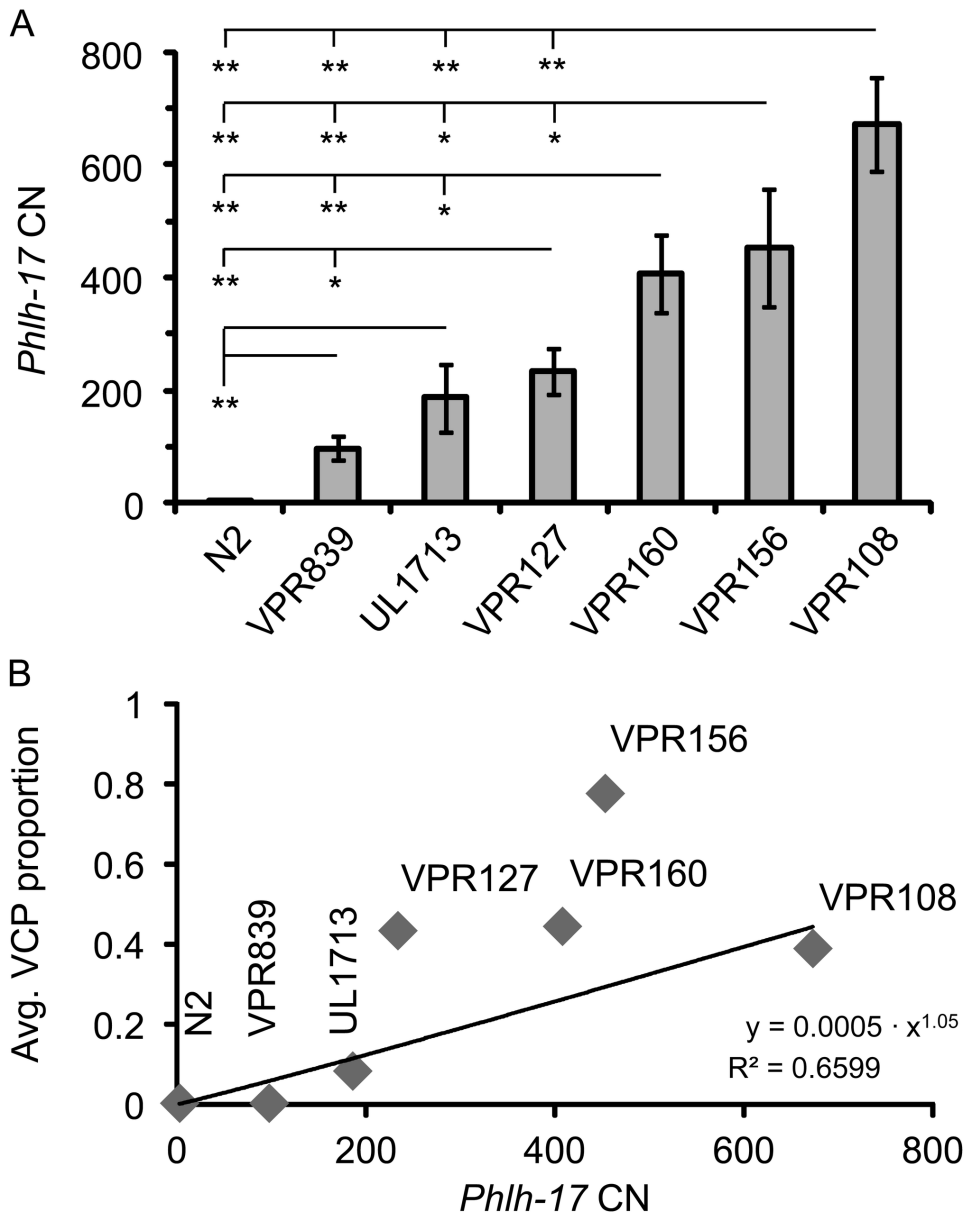
VCP in adult worms is correlated to *Phlh-17* copy number (CN). (A) A *Phlh-17* primer pair was used to determine *Phlh-17* CN using genomic DNA extracts from individual worms (n=6-7 in each group). Bars represent means ± sem. * p<0.05, ** p<0.01; One way ANOVA, followed by Fisher’s least significant difference (LSD) post hoc test. (B) The *Phlh-17* CN (x) and the average VCP proportion (y) show statistically significant correlation (regression ANOVA, p = 0.0264). Equation and the coefficient of determination (R^2^) are shown in the lower right corner.

 We further quantitatively assessed the influence of the t-*Phlh-17* CN on the display of the VCP in adult worms by RT-qPCR (Figure 4). We found that transgenic lines have up to 670 copies of the *Phlh17* (Figure 4 A). The *Phlh-17* CN was generally low for strains that show wild type locomotion and generally high for those displaying the VCP (compare Figure 4 A and Table 2). We found a statistical correlation between the *Phlh17* CN and the average VCP proportion (Figure 4 B), indicating that this behavioral phenotype is transpromoter CN-dependent. 

## Discussion

The results we present here show that a specific locomotion phenotype is caused by transgenic over-representation of the *Phlh-17*. In general this indicates that CN of transgenic arrays is important in research utilizing the t-*Phlh-17* to mark or affect the activities of CEPsh cells. Methods for limiting CN in transgenic worm lines have been developed and may be required depending on how the t-*Phlh-17* is to be used [25-28]. The multiple behavioral tests and CN guidelines established in this work will be useful in determining if a given worm line is acceptable for a specific research application.

Complete loss of *hlh-17* gene expression is lethal and worms hemizygous for the *hlh-17* gene do not show the VCP [10]. Short interfering RNA knockdown of *hlh-17* was not reported to induce the VCP [10,29] indicating: i) that potential silencing of the endogenous *hlh-17* gene product by the t-*Phlh-17* would not cause the VCP, and ii) that while the transcription factor HLH-17 has a role in regulation of its own expression [11], the sequestration of HLH-17 is not likely the cause of the VCP.

Identification of the endogenous factor that is affected by multiple copies of the t-*Phlh-17* is beyond the scope of this study. However, behavioral analysis has been performed by others on a very large number of *C. elegans* mutants and the backwards-only VCP is rare. Unsurprisingly these mutants affect motor neuron development and/or function. One reverse-only VCP-producing gene mutant (*unc-55*) encodes a DNA binding protein and it has homology to vertebrate chicken ovalbumin upstream promoter transcription factor family (COUP-TF) and acts as a repressor [30]. *Unc-55* is required early in development to prevent abnormal synaptic rearrangement of gamma-amino butyric acid (GABA)ergic motor neurons that control body wall muscles [31]. It is very interesting that a mutation eliminating the RNA binding protein MBL-1 leads to a backward VCP in *C. elegans* [32,33]. MBL-1 and its gene *mbl-1* share homology with muscleblind (MBL) of *Drosophila melanogaster* and a human orthologue muscleblind-like (MBNL1) RNA binding proteins. Results of recent work by others in a variety of organisms indicate that some effects of myotonic dystrophy type 1 are mediated by sequestration of MBNL1 by RNA containing expansion of repetitive non-protein-coding sequences, as reviewed in 34, and that MBNL1 can bind to its recognition motif when it is in DNA form [35]. The backward VCP is also seen in an *unc-32* mutation; this gene encodes a vacuolar proton-translocating ATPase subunit that is important for motor neuron signaling and survival [36] in the worm. Splicing of the *unc-32* transcript is regulated by the CELF family UNC-75 [37] and CELF family RNA binding proteins are thought to be involved with aspects of myotonic dystrophy, as reviewed in 34 and references therein. In this case improper splicing of *unc-32* transcripts due to sequestration of UNC-75 might mimic knockout of *unc-32*. Other *C. elegans* genes leading to backwards dorsal coiling or general coiling behavior are also possible candidates for the factor involved in the t-*Phlh-17* induced VCP [38]. 

**Figure 5:**
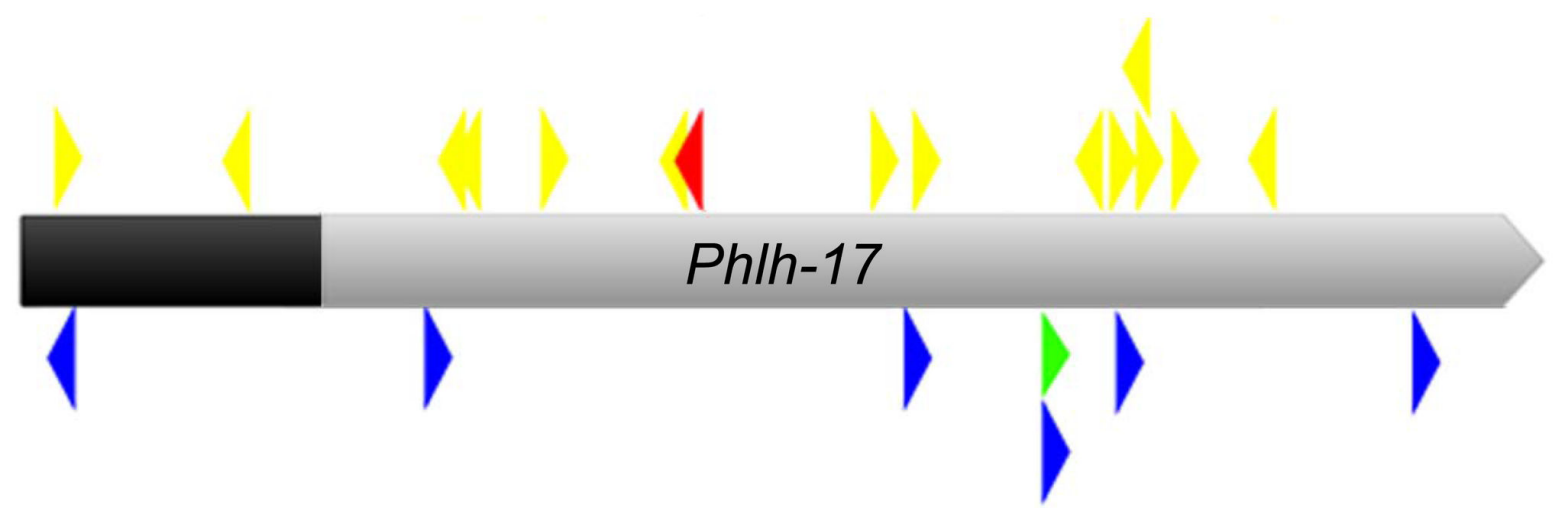
In silico analysis of the *Phlh-17*. Cartoon showing possible binding sites of protein factors associated with backward ventral coiler phenotypes. The 2.5 kbp promoter (full ribbon length) was used in the VPR transgenic strains, while the shorter 2 kbp promoter (gray) was used to make line UL1713. Ribbon is pointing in the direction of transcription. A 100% match for an UNC-55 binding site (half-site for UNC-55 dimer; red arrowhead), along with possible (1 mismatch) other sites for UNC-55 binding (yellow arrowheads), a single CTGCTG site (green arrowhead), and exact site matches for the DNA version of the human MBNL1 binding consensus sequence YGCT(T/G)Y (blue arrowheads) are depicted. Arrowhead direction indicates a match to the top (right facing arrowhead) or bottom (left facing) DNA strand; arrowheads not drawn to scale of binding site relative to the hlh-17 promoter sequence.

In order to do preliminary assessment of what factors may be sequestered to produce the VCP we performed *in silico* analysis of the *Phlh-17* to search for motifs within the sequence representing *cis* binding sites for transcription factors. Several RNA expansion diseases of the human nervous system are caused by repetitive CUG or CCUG sequences in non-coding regions of the genome, as reviewed elsewhere [34]. Therefore, we searched for CTG and CCTG repeats in the *Phlh-17 sequence*. We found only one site for a single repeat of CTG (Figure 5, green arrowhead) and no CCTG repeats, albeit there are clusters of single CTG and CCTG sites throughout the promoter (not shown). We then searched for potential UNC-55 binding sites [39]. We found one site that exactly matches the UNC-55 binding motif (called a half site since UNC-55 binds as a dimer, but can pair with other transcription factors that bind differing sites) at 1.1 kbp from the 5’ end of the t-*Phlh-17* (Figure 5, red arrowhead). We also found 14 half sites with one mismatch from the consensus UNC-55 binding motif including one direct and one palindromic repeat each (Figure 5, yellow arrowheads). Next we used the consensus binding site for human MBNL1 [35,40,41] and found six possible sites scattered throughout the *Phlh-17* (Figure 5, blue arrowheads). The UNC-75 binding motif was recently identified as UUGUUGUGUUGU [37], but the corresponding DNA sequence was not found in the *Phlh-17* even when 2 mismatches were allowed. This suggests that UNC-55 and MBL-1 are good candidates for the factor that is sequestered by the t-*Phlh-17*. Guided by *in silico* promoter analysis, future studies to identify the factor that is sequestered by the t-*Phlh-17* could use over-expression of transcription factors and promoter mutation in combination with the behavior, CN, and breeding methods and results developed for this report. 

*C. elegans* is used extensively to study gene and protein regulatory networks and neurodegenerative diseases. The ease of producing repetitive transgene arrays in *C. elegans* could be used to test the role of RNA/DNA binding protein sequestration to model human repeat expansion-neurodegenerative diseases. In this way the confounding effects of RNA toxicity could be avoided since the transgenes would not require transcription to be present in large repeat-numbers within neural cells of interest.

## Supporting Information

Movie S1 **Normal forward and induced normal backward movement in an adult worm.** The N2 (non-transgenic) worm was induced to crawl backward with tactile stimulus to the anterior region with an eyebrow hair attached to a toothpick. A single trial is shown. Scale bar, 500 µm.
(WMV)

Movie S2 **Normal forward and induced backward ventral coiling in an adult worm.** The VPR156 strain worm was induced to crawl backward with tactile stimulus to the anterior region. A single trial is shown. Scale bar, 500 µm.
(WMV)

Movie S3 **Example of the VCP severity rating 2.** A portion of a video clip used in blind VCP severity rating tests is shown. This worm was a cross between VPR156 and N2 worms (group marked X in Figure 3A), thus heterozygous for the *vprIs156* transgenic array. It was rated 2 (instead of completely wild type=1, as shown in Movie 1) due to the slightly exaggerated bending of the tail in the ventral direction. Scale bar, 500 µm.
(WMV)

Movie S4 **Example of the VCP severity rating 8.** A portion of a video clip used in blind VCP severity rating tests is shown. This was a VPR156 strain worm (homozygous for the *vprIs156* transgenic array). It was rated 8 (instead of completely VCP=10) due its ability to continue to crawl backwards instead of forming a tight coil (as shown in Movie S2). Scale bar, 500 µm.
(WMV)
